# Stimuli-responsive polymeric nanomaterials for rheumatoid arthritis therapy

**DOI:** 10.1007/s41048-020-00117-8

**Published:** 2020-10-31

**Authors:** Yingsi Xie, Ruslan G. Tuguntaev, Cong Mao, Haoting Chen, Ying Tao, Shixiang Wang, Bin Yang, Weisheng Guo

**Affiliations:** 1 Translational Medicine Center, Key Laboratory of Molecular Target & Clinical Pharmacology, School of Pharmaceutical Sciences & the Second Affiliated Hospital, Guangzhou Medical University, Guangzhou 510260, China; 2 The Sixth Affiliated Hospital; Department of Biomedical Engineering, School of Basic Medical Sciences, Guangzhou Medical University, Guangzhou 511436, China; 3 Université Paris 13, 99 avenue Jean Baptiste Clément, 93430 Villetaneuse, France; 4 Department of cardiovascular medicine, the Third Affiliated Hospital, Guangzhou Medical University, Guangzhou 510260, China

**Keywords:** Rheumatoid arthritis, Nanotechnology, Stimuli-responsive polymers, Drug delivery systems

## Abstract

Rheumatoid arthritis (RA) is a long-term inflammatory disease derived from an autoimmune disorder of the synovial membrane. Current therapeutic strategies for RA mainly aim to hamper the macrophages' proliferation and reduce the production of pro-inflammatory cytokines. Therefore, the accumulation of therapeutic agents targeted at the inflammatory site should be a crucial therapeutic strategy. Nowadays, the nanocarrier system incorporated with stimuli-responsive property is being intensively studied, showing the potentially tremendous value of specific therapy. Stimuli-responsive (*i.e*., pH, temperature, light, redox, and enzyme) polymeric nanomaterials, as an important component of nanoparticulate carriers, have been intensively developed for various diseases treatment. A survey of the literature suggests that the use of targeted nanocarriers to deliver therapeutic agents (nanotherapeutics) in the treatment of inflammatory arthritis remains largely unexplored. The lack of suitable stimuli-sensitive polymeric nanomaterials is one of the limitations. Herein, we provide an overview of drug delivery systems prepared from commonly used stimuli-sensitive polymeric nanomaterials and some inorganic agents that have potential in the treatment of RA. The current situation and challenges are also discussed to stimulate a novel thinking about the development of nanomedicine.

## INTRODUCTION

Rheumatoid arthritis (RA) is a long-term inflammatory disease derived from an autoimmune disorder of the synovial membrane (McInnes and O'Dell 2010; McInnes and Schett 2017; Smolen *et al*. 2012). It causes clinically acute pain, swelling, progressive cartilage damage, and bone erosion, which severely affects life quality, even increases the mortality rate (Oliveira *et al*. 2018). While the cause of rheumatoid arthritis is not very clear, the underlying mechanism of pathogenesis involves the immune system attacking the joints. The major evidence from genetics, tissue analyses, and clinical studies points to an immune-mediated etiology associated with stromal tissue dysregulation that together propagate chronic inflammation and articular destruction. A pre-RA phase can last months to years in patients. It may alter metabolism by the presence of circulating autoantibodies, which increases concentration and range of inflammatory cytokines and chemokines (Firestein and McInnes 2017). Studies over the last several decades have identified that macrophages play a crucial role in the occurrence and perpetuation of RA. Macrophages are activated by a combination of genetic and environmental factors, of these, the strongest associations have been seen with female sex, a family history of RA, the genetic factor, the shared epitope, and exposure to tobacco smoke (Deane *et al*. 2017). When macrophages are activated, a variety of pro-inflammatory cytokines (tumor necrosis factor-alpha (TNF-α), interleukin-1 beta (IL-1β), IL-6, *etc*.) are excessively secreted in synovial fluid and evoke inflammation and joint destruction (Aletaha and Smolen 2018; Jain *et al*. 2015; Pham 2011). Animal models provide further evidence of the importance of these cytokines in RA development mechanism. Mice expressing a dysregulated and modified human TNF transgene developed spontaneous arthritis. A monoclonal antibody against human TNF-α as a therapeutic approach can completely inhibit the development of this disease (Keffer *et al*. 1991). Hence, current therapeutic strategies for RA are mainly aiming to reduce the production of pro-inflammatory cytokines (Lima and Reis 2015).

RA therapy is complex and includes various classes of medications with different mechanisms of action. Conventional drugs for RA treatment generally fall into four groups: non-steroidal anti-inflammatory drugs (NSAIDs) (Sostres *et al*. 2010), glucocorticoids (GCs) (Smolen *et al*. 2018; Vandewalle *et al*. 2018), non-biological disease-modifying anti-rheumatic drugs (DMARDs), and biological DMARDs (Quan *et al*. 2008). However, RA remains a non-curable disease, its treatment aims to relieve pain and to impede the inflammatory and destructive processes, and the final goal is to achieve remission or at least low disease activity. Such a strategy requires frequent and long-term treatments that can cause undesirable systemic side effects due to the lack of tissue specificity and inability to selectively target the inflammatory areas. Short biological half-life and low bioavailability of conventional drugs also contribute to a low therapeutic index (Guo *et al*. 2018). These limitations have driven the development and application of nanomaterial-based devices in the treatment of RA.

**Figure 1:**
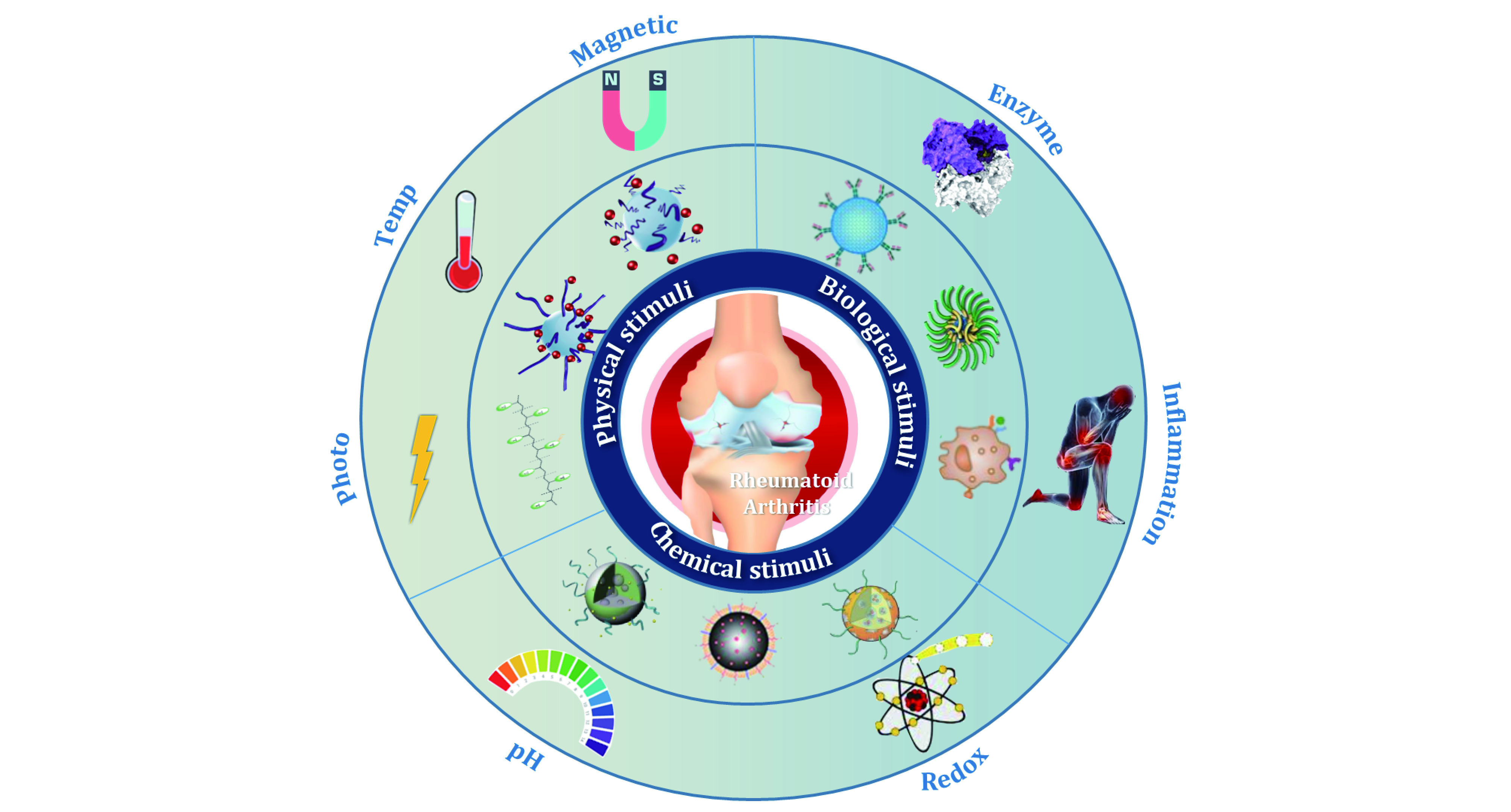
Schematic illustration of stimuli-responsive polymers for rheumatoid arthritis therapy

**Figure 2:**
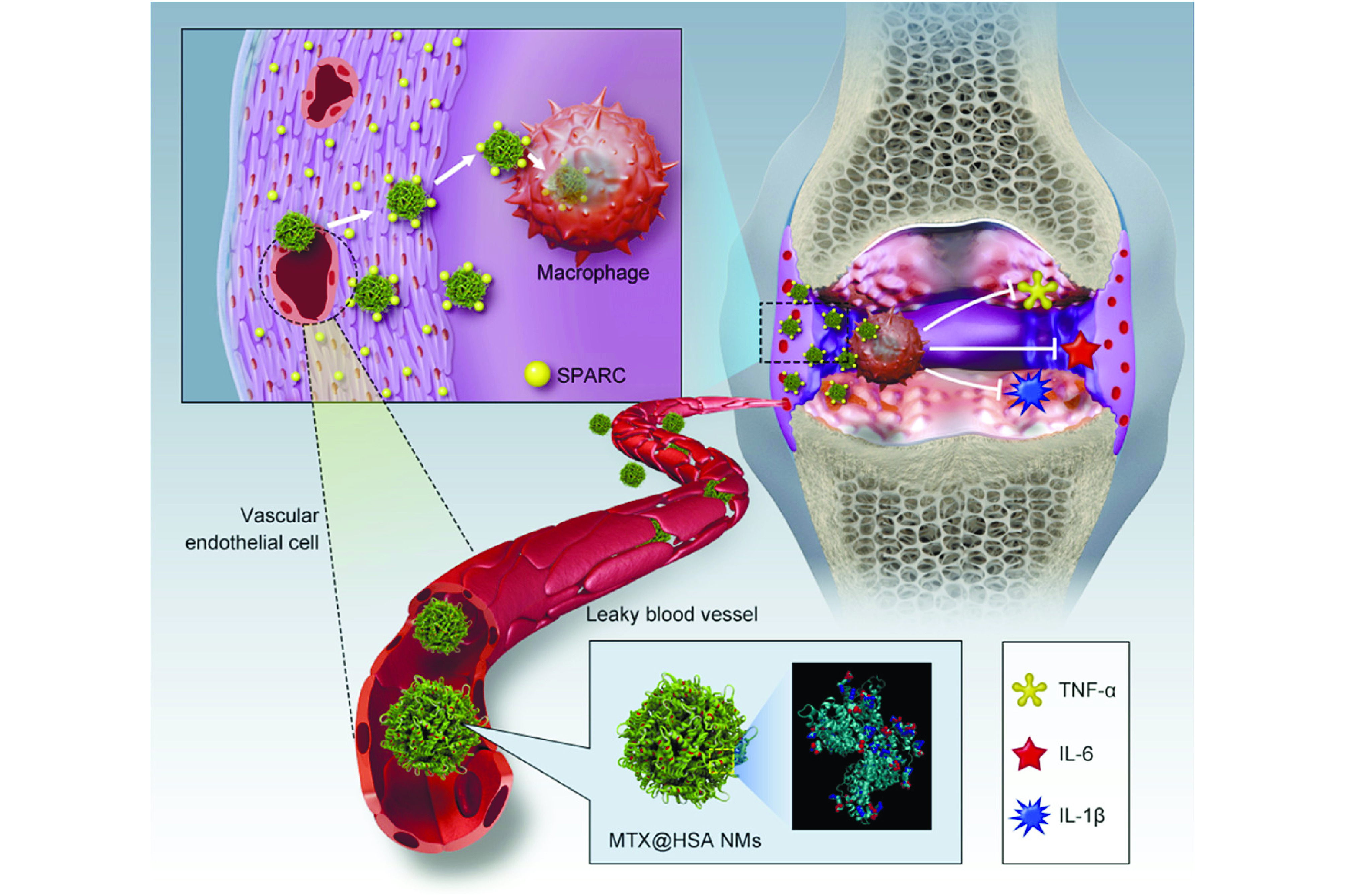
Schematic representation of delivery of MTX@HSA NMs in RA joints. High accumulation of MTX@HSA NMs in the synovium is provided by leaky vessels, enhanced demand for albumin of arthritic joints, and high affinity between HAS and SPARC. After that, activated macrophages take up the MTX@HSA NMs that subsequently inhibit the secretion of pro-inflammatory cytokines, resulting in attenuation of RA (Liu *et al*. 2019)

Nanomedicines (NMs) offer multiple benefits over conventional drugs in RA treatment. Above all, their site-specific and target-oriented drug delivery is demonstrated thanks to nanoparticles’ detection and responses to stimuli from the micro- or macroenvironment. Depending on the nature of the process, the responsible stimuli can be divided into three groups: physical (*e.g*., temperature, light, magnetic/electric field), chemical (*e.g*., pH, redox), and biological (*e.g*., enzymes, endogenous receptors) (Indermun *et al*. 2018; Lopes *et al*. 2013) (Fig. 1). Besides, long circulation time and high bioavailability are their important advantages, due to their appropriate size and easily modified surface (Patra *et al*. 2018). Recently, NMs have been demonstrated as an effective approach for specific drug delivery in the inflammatory tissues. Encapsulation of therapeutic drugs into a nanosized drug delivery system allowing accurate drug delivery can increase drug absorption and biodistribution in affected sites resulting in enhanced therapeutic efficacy. Furthermore, surface functionalization or surface coating is considered as a crucial parameter for improving the targeting efficiency and can increase cellular uptake. For instance, folic acid, a stable and non-immunogenic ligand that has a high affinity to cell surface folate receptor (FR), was employed as a targeting moiety of the dendrimer to attain specific targeting since FR is overexpressed on activated (but not quiescent) macrophage (Nogueira *et al*. 2016). Liu *et al*. reported another example of specific delivery of anti-rheumatic drugs without using targeting ligands (Liu *et al*. 2019). The authors found overexpression of SPARC (secreted protein acidic and rich in cysteine) in the synovial fluid and synovium during RA. Motivated by the high affinity of SPARC for albumin, they developed methotrexate (MTX)-loaded human serum albumin (HSA) NMs (MTX@HSA NMs) for biomimetic drug delivery (Fig. 2). Besides, since arthritic joints consume higher amounts of albumin than healthy tissues, increased demand for albumin by arthritic joints can contribute to specific drug delivery. The fabricated nanotherapeutics were intravenously injected into mice with collagen-induced arthritis (CIA). The fluorescence/magnetic resonance dual-modal imaging analysis displayed a higher accumulation and prolonged retention time of MTX@HSA NMs in arthritic joints as compared to free HSA and MTX molecules. *In vivo* evaluations evidenced that unlike free MTX, the MTX@HSA NMs had a superior therapeutic index and fewer side effects. Moreover, the results also demonstrated higher RA treatment efficacy of nanoformulation even at a half dose of administrated MTX for free MTX.

Site-specific delivery of the drugs can also be achieved by using “smart” NMs fabricated of stimuli-responsive materials that can recognize microenvironment and response in a dynamic way. Application of stimuli is responsible for alteration/disruption of the structure of specifically designed nanocarriers, resulting in drug release at the targeted site (Stuart *et al*. 2010). The advances in the past 10 years on the use of stimuli-responsive polymeric nanoparticles for the treatment of RA were summarized in Table 1.

**Table 1:** The published articles on the use of stimuli-responsive polymeric nanoparticles for the treatment of RA

Type of stimuli	Stimulus	Description of the system	Drug	Reference
Chemical	pH	Mineralized nanoparticles composed of PEGylated hyaluronic acid (P-HA) as the hydrophilic shell, 5β-cholanic acid as the hydrophobic core, and CaP as the pH-responsive mineral	ΜΤΧ	Alam *et al*. 2017
		Hyaluronic acid (HA) coated acid-sensitive polymeric nanoparticles (HAPNPs) composed of egg phosphatidylcholine, polyethylenimine, and PCADK	Dex	Yu *et al*. 2019
		Stearic acid-octa-arginine and folic acid decorated PLGA -PK3-based lipid polymeric hybrid nanoparticles (Sta-R8-FA-PPLPNs/MTX) composed of PK3, Folate-PEG-PLGA, egg PC, and Sta-R8	ΜΤΧ	Zhao *et al*. 2018
		Poly(β-amino ester)-graft-poly(ethylene glycol) (PAE-g-PEG) self-assembled into spherical micelles and MTX loaded into the hydrophobic core of micelles	ΜΤΧ	Moon *et al*. 2020
		Multifunctional FA receptor-targeting and pH-responsive nanocarriers composed of lipids, PEG-PLGA forming a hydrophilic shell, folic acid (FA) around the hydrophilic shell as a targeting ligand, and poly (cyclohexane-1,4-diylacetone dimethylene ketal) (PCADK) and PLGA as a hydrophobic core. PCADK also as a pH-responsive material	ΜΤΧ	Zhao *et al*. 2017
		Modular pH-sensitive acetone-based ketal-linked prodrugs of dexamethasone (AKP-dexs) nanoparticles	Dex	Xu *et al*. 2020
Physical	NIR	RGD-attached gold (Au) half-shell nanoparticles containing methotrexate (MTX), RGD peptide as targeting moiety for inflammation	ΜΤΧ	Lee *et al*. 2013
Biological	Redox (ROS)	Folate conjugated to PEC 100 monostearate as film-forming material, and methotrexate (MTX) and catalase (CAT) co-encapsulated liposomes (FOL-MTX&CAT-L)	ΜΤΧ	Chen *et al*. 2019b
Combined	Temperature and pH	Methotrexate (MTX) and gold (Au) nanoparticles incorporated in the pegylated-poly(_DL_-lactic-co-glycolic acid) nanospheres	MTX	Lima and Reis 2015
	NIR and external magnetic field	MTX-loaded poly(lactic-co-glycolic acid) (PLGA) gold (Au) / iron (Fe) / gold (Au) half-shell nanoparticles conjugated with arginine-glycine-aspartic acid (RGD)	MTX	Kim *et al*. 2015

These triggered systems can be sensitive to various endogenous stimuli, such as lowered interstitial pH, an enhanced concentration of certain enzymes, or a high level of glutathione. At the cellular level, pH-sensitive nanocarriers can either induce the release of the encapsulated drug into late endosomes or lysosomes, or stimulate the escape of nanodevices from the lysosomes to the cell cytoplasm (Karimi *et al*. 2016a). At the tissue level, stimuli-responsive NMs can take advantage of microenvironmental features associated with pathological conditions including neoplastic diseases, inflammation, ischemia, or infections. External stimuli can also be applied. For instance, the specific delivery of therapeutic agents to the desired tissues can be magnetically guided by inorganic agents, such as ultrasmall iron oxide-based nanoparticles. Sustained drug release can also be achieved by light-, thermo-, or ultrasound-sensitive nanocarriers (Mura *et al*. 2013; Raza *et al*. 2019).

The fabrication of stimuli-responsive nanocarriers requires the use of biocompatible materials, which are able to undergo supramolecular conformational changes, specific protonation, hydrolytic cleavage, *etc*. The most appropriate and widely explored category of materials that possess a potentially tremendous value of specific therapy is polymers of both natural and synthetic origin. The versatility of polymer sources and the ability of their combinatorial synthesis make it possible to control polymer sensitivity to a certain stimulus within a narrow range (James *et al*. 2014). Moreover, ease of polymers modification can lead to increased biocompatibility and reduced toxicity. Many synthetic stimuli-responsive polymers including thermo-responsive pluronic F127 (PF127), which is a non-ionic triblock copolymer of poly(ethylene oxide)-b-poly(propylene oxide)-b-poly(ethylene oxide) (PEO-PPO-PEO), or poly(*N*-isopoprylacrilamide) (pNIPAAm) had been approved by U.S. Food and Drug Administration (FDA) for biomedical applications, especially for drug delivery and tissue engineering (Chatterjee and Hui 2019). In this review, we focused on the latest progress of stimuli-responsive polymeric NMs for RA therapy. Some existing studies and thinking on NMs are also discussed.

## STIMULI-RESPONSIVE POLYMERIC NANOMATERIALS

**Figure 3:**
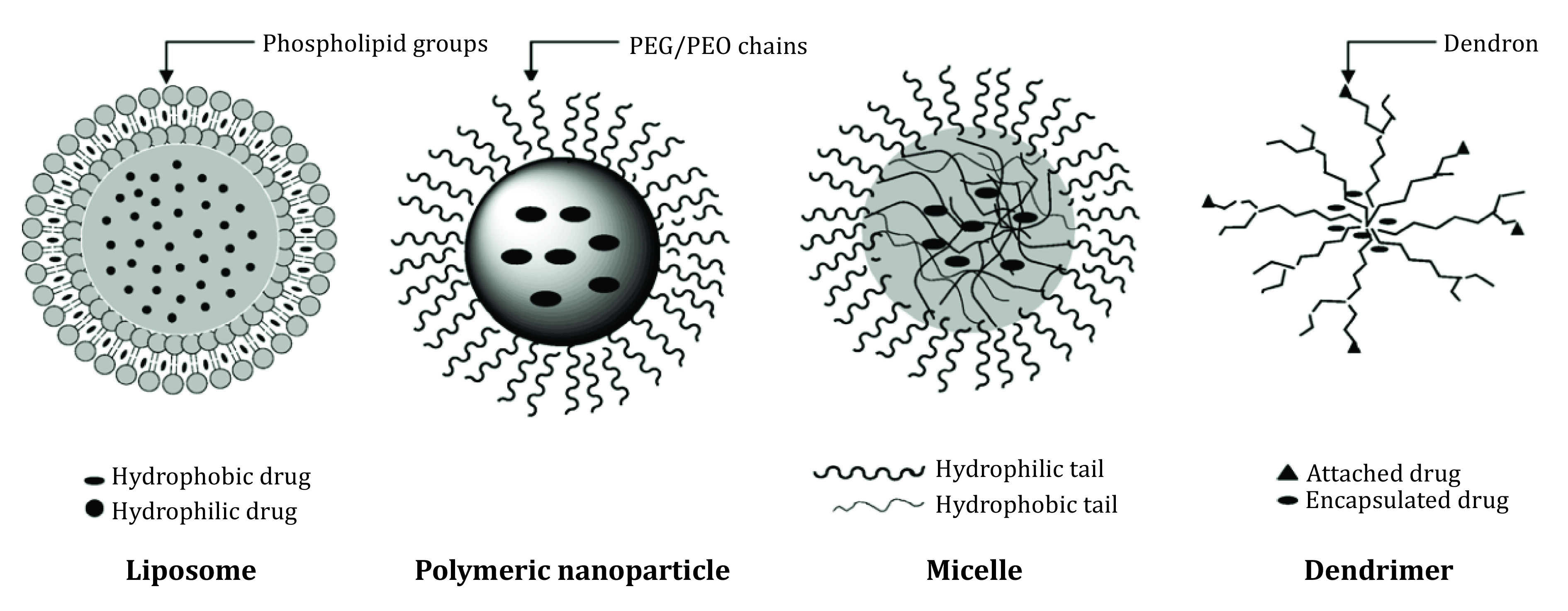
Schematic representation of different types of stimuli-responsive nanocarriers (Ganta *et al*. 2008)

Polymeric materials are one of the most commonly used building blocks for the preparation of drug delivery systems. They are becoming increasingly important in the biomedical field since they offer several advantages, as compared to other materials, such as high synthetic flexibility, ability to be precisely tailored according to their specific application, biological functionality, biodegradation, biocompatibility, and suitable mechanical properties (Song *et al*. 2018). Stimuli-sensitive polymers undergo an abrupt change in their physical properties in response to various stimuli from the microenvironment or macroenvironment (Siafaka *et al*. 2016; Yang *et al*. 2014). The main polymer transitions can be attributed to changes in shape, physical state, solubility, hydrophilic/lipophilic balances, solvent interactions, and conductivity. The driving forces behind these alterations include simple chemical reactions such as reduction–oxidation, acid-base reaction, or hydrolysis of moieties linked to the polymer chain. Some of the changes are reversible because of the polymers’ ability to return to the original state after the trigger is removed. In some cases, external stimuli cause irreversible bond breakage leading to degradation and dramatic conformational change in the polymeric structure (James *et al*. 2014). Polymeric nanoparticles (PNPs) account for a great proportion in stimuli-responsive nanocarriers including nanocapsules, nanospheres, liposomes, polymeric micelles, dendrimers, *etc*. (Fig. 3). The major benefits of sensitive drug delivery systems include accurate drug delivery, controlled payload release, increased duration time of therapeutic agents in the body, improved stability, and reduced dosing frequency. These benefits can ultimately improve the therapeutic index and reduce systemic side effects of conventionally used drugs. Novel strategies for RA treatment are usually modeled after oncology and apply the same polymeric materials with or without structural changes that are adopted for the specific features of RA. Herein, we introduce strategies of RA treatment that take advantage of applying stimuli-sensitive PNPs.

### pH-responsive PNPs

pH shift has been exploited to trigger the release of incorporated therapeutics when environmental alterations are associated with pathological conditions, such as inflammation (Karimi *et al*. 2016a). It is well known, that the normal knee-joints have a physiological pH of 7.3, while the pH value of rheumatoid inflammatory tissues is intrinsically acidic (pH 6.6) (Goldie and Nachemson 1969). The drop in pH is related to infiltration and activation of inflammatory cells in the synovial tissues, resulting in enhanced energy and oxygen demand. Accelerated glucose consumption via glycolysis results in increased lactic acid production, which leads to the local acidosis (Borregaard and Herlin 1982; Menkin 1956; Rajamäki *et al*. 2013; Roiniotis *et al*. 2009). Importantly, in RA patients, the low synovial pH correlates with high disease activity and joint destruction (Farr *et al*. 1985; Geborek *et al*. 1989). In this regard, the strategy of using pH-sensitive PNPs bearing therapeutic agents (NSAIDs, GCs) has great potential in RA treatment. The nanosystems with an appropriate size can effectively penetrate inflamed joints by taking advantage of enhanced angiogenesis during RA, which leads to discontinuity of inflammatory endothelial cells and increased vascular permeability (Peper *et al*. 2017; Wang *et al*. 2020). After sensitive nanocarriers have delivered anti-RA drugs to the affected area, low pH conditions trigger the drug release precisely into its site of action. Applying this method makes it possible to enhance the efficacy of RA treatment by improving therapeutic selectivity and reducing systemic side effects.

The rational design of pH-sensitive nanoparticles is based on two main strategies: the use of polymeric carriers with acid-sensitive bonds, which under low pH conditions are cleaved, resulting in the release of molecules anchored at the polymer backbone; and the use of polymers (polybases or polyacids) with ionizable groups that undergo a pH-dependent conformational or solubility changes (Murthy *et al*. 2003; Yang *et al*. 2014). Some of the pH-sensitive polymers employed in the field of nanomedicine are summarized in Table 2.

**Table 2:** Examples of pH-sensitive polymeric materials which can be applied for RA therapy

Polymeric material	Size of nanoparticles (nm)	Stimulus	Response pH value	Reference
Poly(2-tetrahydropyranyl methacrylate)	314	pH (acetal bone)	5.1	Jung *et al*. 2007
Poly(ethylene oxide)–poly(β-amino ester)	150–200	pH (dissolution mechanism)	< 6.5	Shenoy *et al*. 2005
Poly(ethylenimine)–poly(methacryloyl sulfadimethoxine) (PSD)-block-PEG	300	pH (sulfonamide group)	6.6	Sethuraman *et al*. 2006
Poly(ethylene glycol)-poly (_L_-histidine)-poly(_L_-lactide)	803	pH (imidazole group)	5.0	Liu *et al*. 2011
Chitosan–histidine–arginine	105	pH (imidazole group)	6–5	Gaspar *et al*. 2013
(5-methyl-2-(2,4,6-trimethoxyphenyl)-[1,3]-5-dioxanylmethyl methacrylate) and (1,4-O-methacryloylhydroquinone)	20–100	pH (acetal bone)	5	Zubris *et al*. 2013
PEG–aldehyde functionalized dextran–DOX	100	pH (imine bonds)	5.5	Sagnella *et al*. 2014
Poly(oligoethylene glycol) methyl ether acrylate	10–20	pH (imine bonds)	5.5	Liu *et al*. 2012
Poly(oligoethylene methoxy acrylate) modified with NO donor molecules	32	pH	< 7.0	Duong *et al*. 2014
Poly(lactic-co-glycolic acid) and eudragit S-100	250	pH (dissolution mechanism)	> 7.57	Zhang *et al*. 2011

**Figure 4:**
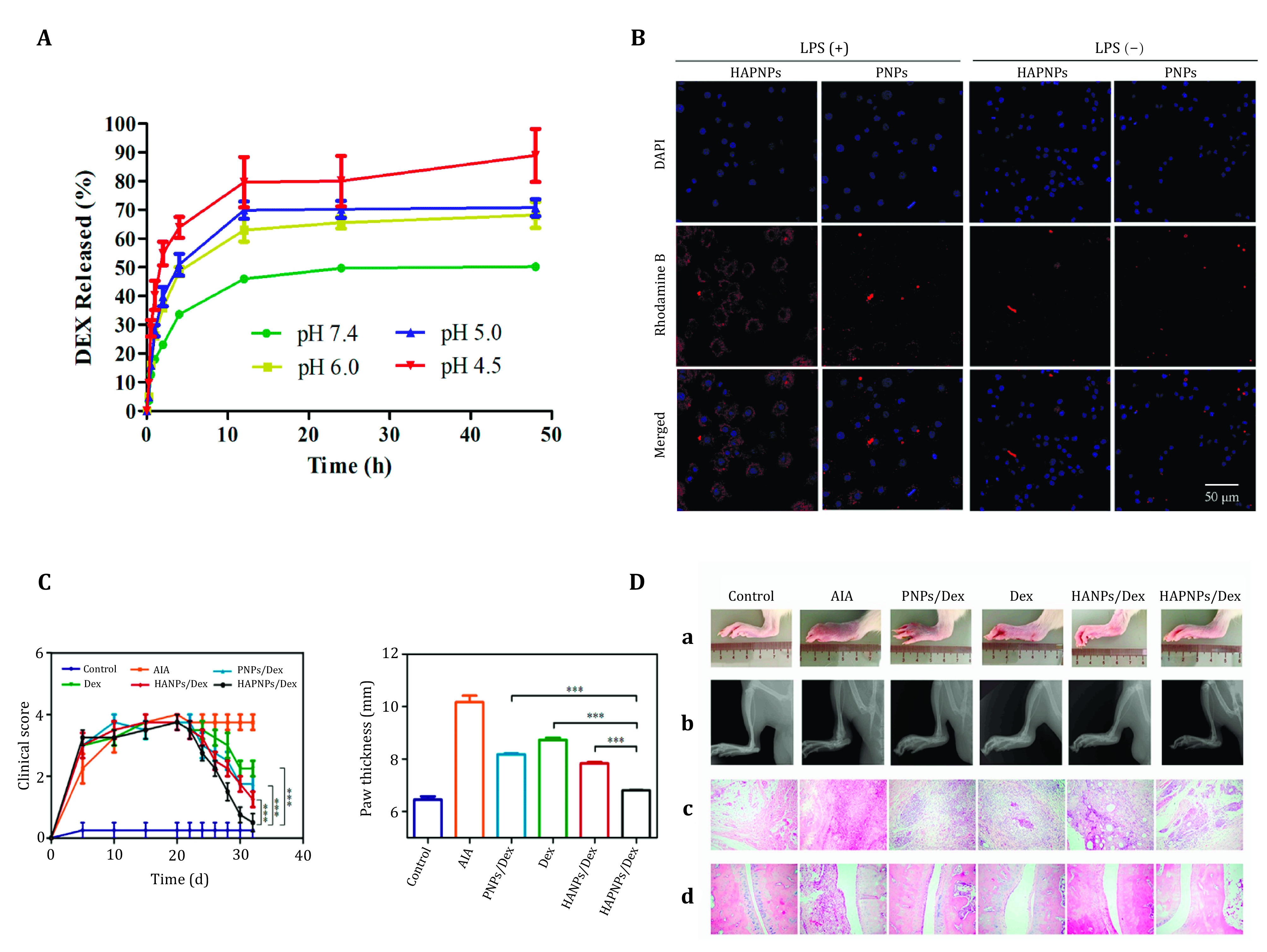
Experimental outcomes of HAPNPs applied for the treatment of RA. **A** *In vitro* drug release profile of HAPNPs/Dex. **B** Confocal laser scanning microscope images of RAW 264.7 cells after activation by LPS (+) or not (−) incubated with rhodamine B-loaded PNPs and HAPNPs.**C** Clinical score of RA as a function of days after incubation and hind paw thickness of AIA rats on day 32 after induction. Results presented as mean ± SD (*n* = 6, ***p < 0.001). **D** Therapeutic effects of HAPNPs/Dex, PNPs/Dex, HANPs/Dex and free Dex in AIA rats. **a** Photographs of AIA rat paws from the different groups. **b** Hind paws of AIA rats imaged by X-ray. **c** Periarticular soft tissues of hind paws histologically identified by H&E staining.**d** Ankle joints of hind paws in the different group histologically identified by H&E staining (Yu*et al*. 2019)

Researchers have designed new approaches to attain targeted release by nanocarrier surface functionality and coating. It was demonstrated that the strategy of simultaneous use of pH-responsiveness and active targeting could markedly improve therapeutic specificity in RA treatment. Yu *et al*. investigated a modification of hyaluronic acid (HA) coated acid-sensitive PNPs (HAPNPs) loaded with dexamethasone (Dex) for the treatment of RA (Yu *et al*. 2019). The acid-sensitive polymeric material is composed of egg phosphatidylcholine, polyethylenimine, poly(cyclohexane-1,4-diyl acetone dimethylene ketal) (PCADK), a member of the polyketal family, which has excellent acid sensitivity. HA was selected as a targeting moiety due to its ability to bind CD44, an adhesion receptor, which is overexpressed on the surface of activated macrophages in RA patients. It was verified, that the fabricated nanosystem with an average diameter of 150.5 nm, possessed a marked pH-dependent drug release behavior. The presence of HA on the surface of nanoparticles enabled high targeting ability of HAPNPs towards activated macrophages, which was confirmed by cellular uptake studies using confocal laser scanning microscopy. Finally, *in vivo* experiments in adjuvant-induced arthritis (AIA) rat models showed that the inflammatory cell infiltration, bone damage, and cartilage damage were reduced in the ankle joints under the treatment by HAPNPs/Dex. An increased curative effect was attained most likely due to the pH sensitivity of HAPNPs and their targeting ability to the activated macrophages (Fig. 4).

**Figure 5:**
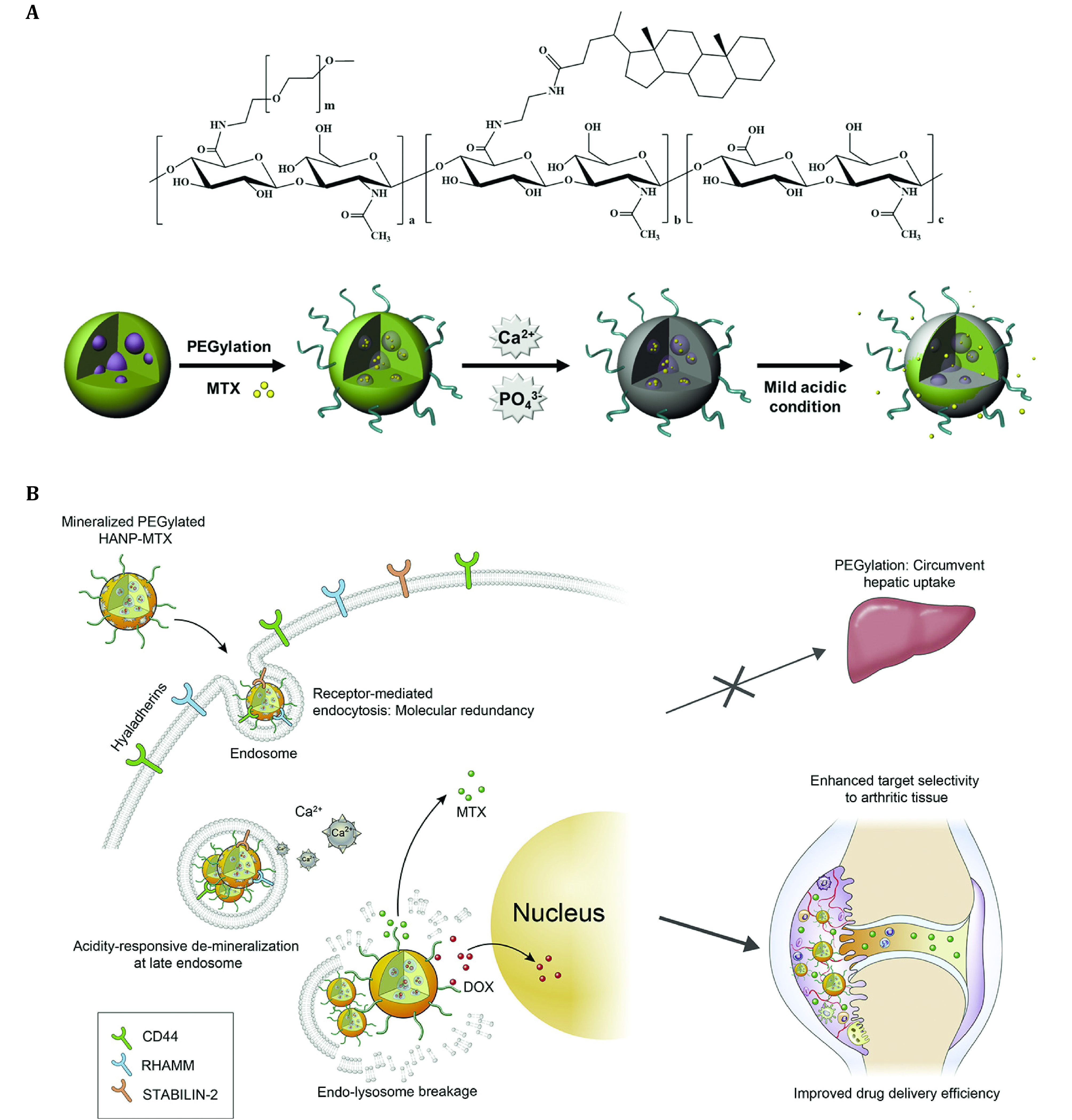
Synthesis and application of mineralized PEGylated HANP-MTX. **A** Chemical structure of PEGylated HANPs and diagram showing MTX loading of PEGylated HANPs, followed by calcium phosphate mineralization, which is demineralized and released the drug in the acidic environments. **B** Schematic presentation of the working mechanism of mineralized PEGylated hyaluronan nanoparticles (MP-HANPs) loaded with methotrexate (MTX) or doxorubicin (DOX) (Alam *et al*. 2017)

Despite polymeric materials, inorganic components can also be applied to provide pH-responsible behavior of the fabricated nanosystem. Such a system was developed by Alam *et al*. (Alam *et al*. 2017). They reported mineralized nanoparticles (MP-HANPs) composed of PEGylated hyaluronic acid (P-HA) as the hydrophilic shell, 5β-cholanic acid as the hydrophobic core, with a calciumphosphate (CaP) as the pH-responsive mineral (Fig. 5A). The presence of CaP as the diffusion barrier enabled pH-responsive release kinetics of incorporated MTX across neutral to acidic conditions. HANPs were internalized in macrophages via receptor-mediated endocytosis, and later MP-HANPs loaded with doxorubicin demonstrated a pH-dependent payload release into the cytosol (Fig. 5B). Further animal studies showed a substantially high paw-to-liver ratio of fluorescent intensity after injection of MP-HANPs loaded with Cy5.5, suggesting improved biodistribution of prepared drug delivery system. Treatment with MTX-loaded MP-HANPs alleviated progression CIA in mice with high safety even when using high doses of MTX.

**Figure 6:**
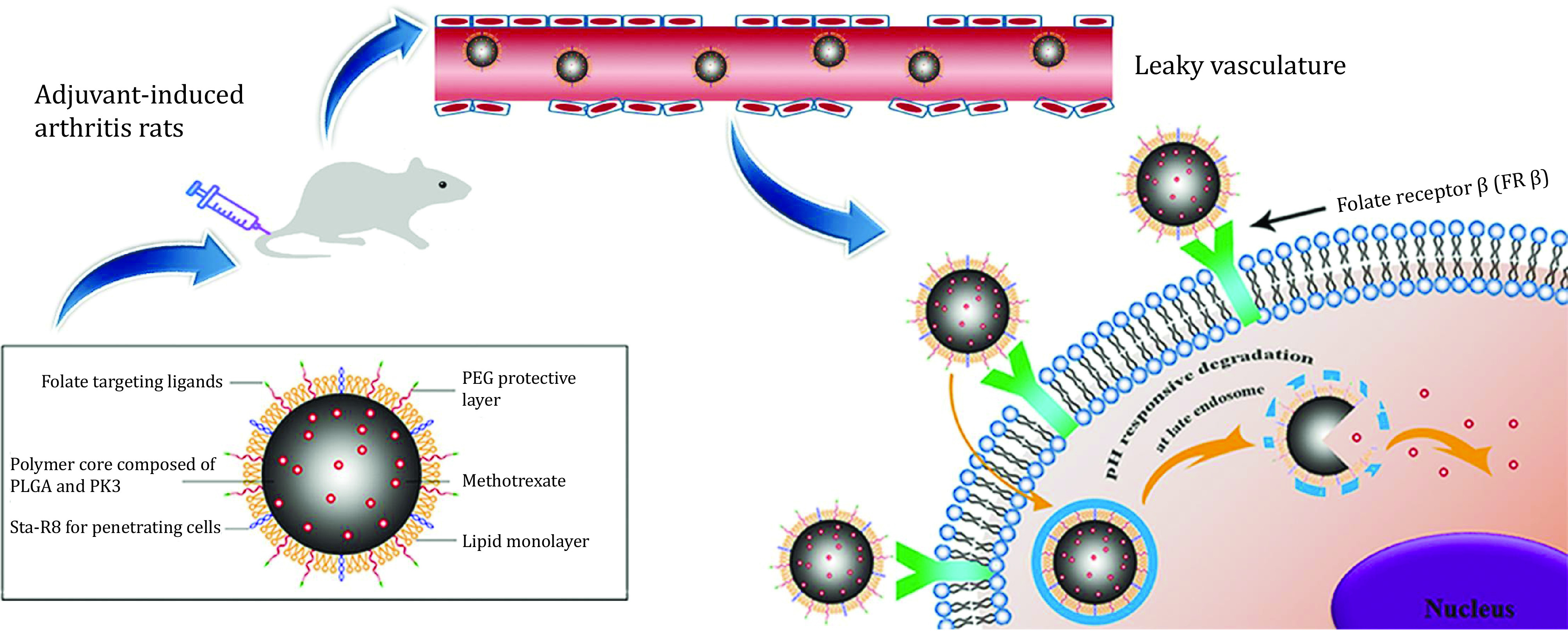
Schematic presentation of the working mechanism of dual-functional lipid polymeric hybrid pH-responsive nanoparticles decorated with cell-penetrating peptide and folate for therapy against rheumatoid arthritis (Zhao *et al*. 2018)

In some cases, pH-responsible drug release can be achieved by the fabrication of nanocarrier’s core through the simple physical mixing of acid-sensitive and non-sensitive polymers. For instance, Zhao *et al*. designed dual-functional lipid polymeric hybrid pH-responsive nanoparticles against RA (Zhao *et al*. 2018). The polymeric core of the system was composed of pH-responsible polyketal (PK3) and conventional PEG-PLGA building blocks. Moreover, the nanoparticles were decorated with folate targeting ligand and hydrophobic derivatives of octa-arginine with stearic acids (Sta-R8) for penetrating macrophages (Fig. 6). MTX was used as an anti-RA drug. The obtained data showed that *in vitro* release of MTX from formulated particles (size 100–150 nm) was faster under acidic conditions (pH 5.0) as compared to physiological pH of 7.4, suggesting apparent pH-responsive characteristic of the nanocarrier. The cellular studies revealed that the presence of folate and Sta-R8 on the surface of nanoparticles improved their uptake efficiency and cytotoxicity. Further *in vivo* therapeutic effects studies indicated that fabricated nanosystem has good therapeutic effects for AIA on rats due to inhibiting the secretion of pro-inflammatory cytokines.

**Figure 7:**
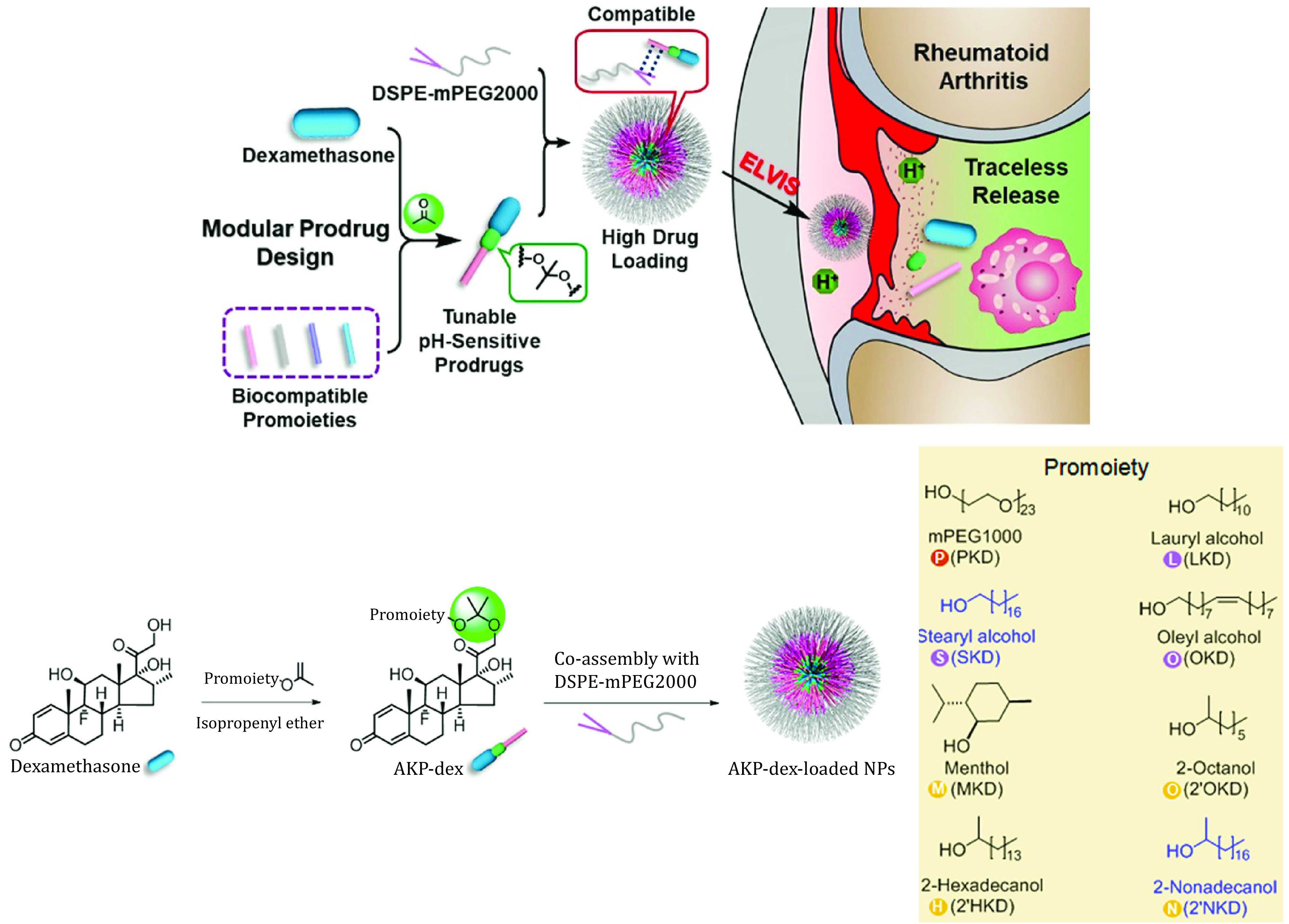
Schematic representation of Dex-loaded nanoparticles mechanism of action and synthesis route of prodrugs (Xu *et al*. 2020)

The traditional design of stimuli-responsive NMs is based on the incorporation of drug molecules into the nanocarrier, which is prepared from pH-sensitive building blocks. However, under acidic conditions, drug delivery systems tend to demonstrate burst drug release instead of extended-release. Moreover, the delivery systems also suffer from the lack of adequate drug loading capacity. The reasons of these phenomena might be attributed to the poor compatibility between encapsulated cargo and nanocarriers (van der Meel *et al*. 2019). In this regard, it becomes pivotal to initiate the finding of innovative strategies to optimize the design of stimuli-responsive NMs. Xu *et al*. proposed an interesting solution to this issue (Xu *et al*. 2020). They developed a Dex prodrug, which was linked via pH-sensitive acetone-based ketals with differently structured pro-moieties. The prodrugs then were loaded into FDA-approved 1,2-distearoyl-sn-glycero-3-phosphoethanol-amine-poly(ethylene glycol) (DSPE-mPEG2000) to formulate NMs for RA treatment (Fig. 7). Better compatibility between Dex and DSPE-mPEG2000, and thus, good stability and high encapsulation efficiency, were achieved by modifying the drug with long-carbon-chain alcohols as pro-moieties: stearyl alcohol and 2-nonadecanol. The synthesized Dex prodrugs were indicated as stearyl alcohol-ketal-Dex (SKD) and 2-nonadecanol-ketal-Dex (2’NKD). SKD-loaded DSPE-mPEG2000 nanoparticles and 2’NKD-loaded DSPE-mPEG2000 nanoparticles were used for further evaluations. The nanoparticles with the mean particle sizes of 10–200 nm demonstrated an effective ability to target the inflamed synovium via extravasation through leaky vasculature and subsequent inflammatory cell-mediated sequestration (ELVIS) effect. A pH-dependent payload release from the nanocarrier was detected in activated macrophages due to ketal hydrolysis under acidic conditions of lysosomes. Nanoparticles also considerably prolonged Dex circulation in plasma in comparison to free Dex sodium phosphate (DSP) that is widely used in the clinic. The AUC_0-∞_ of total Dex from SKD-loaded nanoparticles and 2’NKD-loaded nanoparticles were 17.7-fold and 9.7-fold higher, respectively than that of free DSP. Evaluations of *in vivo* therapeutic efficacy of nanoparticles revealed a substantial anti-inflammation effect because of inhibition of inflammatory cytokines (TNF-α and IL-1β), which ultimately, led to cartilage preservation and prevented bone destruction.

### Temperature-responsive PNPs

**Figure 8:**
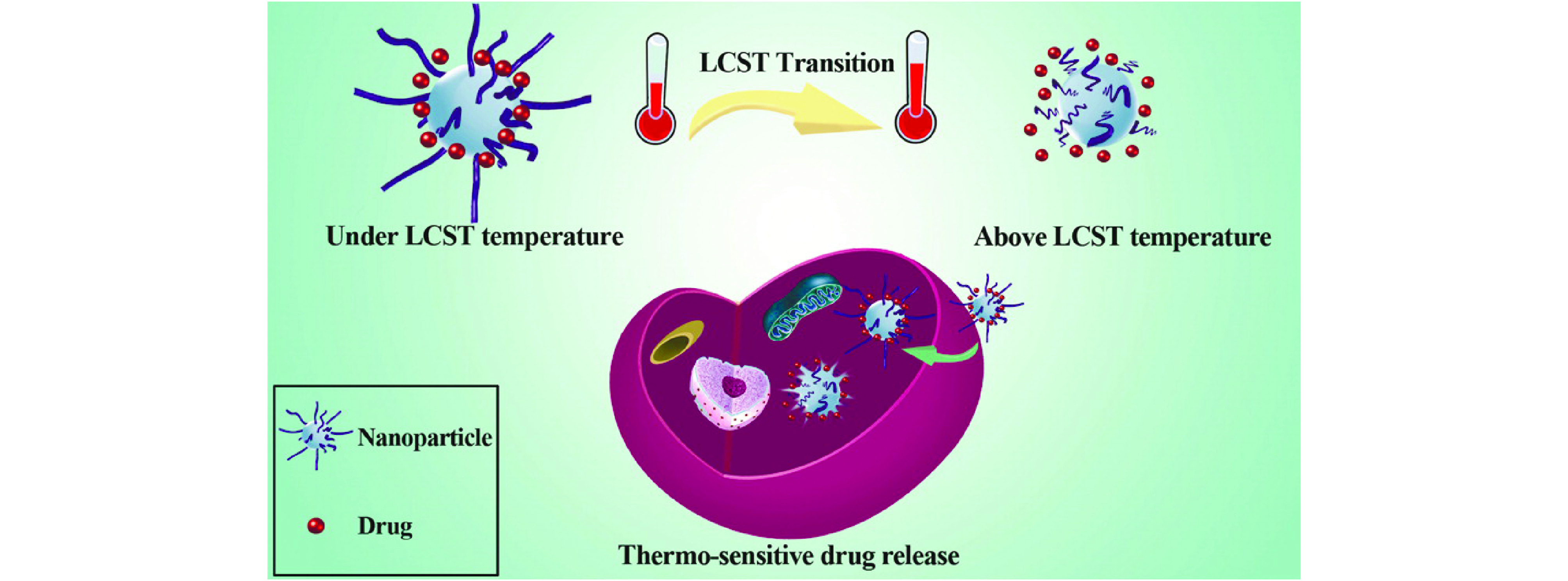
Mechanism of action of thermo-responsive drug delivery system (Karimi *et al*. 2016b)

Temperature-responsive polymers are designed based on their phase-transition behavior in response to changes in temperature. Thermo-responsive behavior of nanocarriers is usually achieved by applying the lower critical solution temperature (LCST) polymers whose solubility behavior is controlled by temperature alterations. LCST is the temperature, below which the components of a mixture are completely miscible in all proportions. Below LCST, the solubility of polymers with transitional behavior is increased and polymeric components are swollen because of hydrogen bonds formed between water molecules and the polymer functional groups, making them ready to be loaded with drug molecules. Once the temperature is slightly above the LCST, a hydrophilic-hydrophobic transition takes place, and it accompanied by a morphological transition from coil-to-globule. During this change, the hydrogen bonds and the network collapse, and the polymer becomes insoluble, resulting in volumetric shrinkage and squeezing-out of internal water molecules (Karimi *et al*. 2016b). This transition triggers the release of the encapsulated guest drug molecules (Fig. 8).

Nowadays, hyperthermia is a method applied in the treatment of many diseases, including RA. The idea of the utilization of thermo-sensitive systems for biomedical applications is from the temperature difference between pathological and healthy tissues (Singh and Amiji 2018). Thermal energy can be provided directly, or external heat sources such as NIR are applied indirectly in RA, which both arouses a response of thermo-sensitive nanomaterials. Usually, the range of temperatures employed is within the 38.5–43°C (Karimi *et al*. 2016a). Stimuli can come from the human body, and local hyperthermia, or hypothermia may arouse a response of thermo-sensitive nanomaterials. Thermo-responsive drug delivery systems can also be designed according to the required application, for instance, to achieve temperature-mediated drug release with the accumulation of drugs at the desired biological area. The other advantages of thermo-sensitive polymeric carriers can be attributed to avoidance of using toxic organic solvents during the preparation process, the ability to encapsulate both hydrophilic and lipophilic drug molecules, and sustained release properties. Multiple polymers have been demonstrated for the fabrication of temperature-responsive systems, including poly(N-isopropylacrylamide (PNIPAAm) derivatives, poly(N-vinylcaprolactam), pluronics (poly(ethylene oxide)-poly(propylene oxide) (PEO-PPO)), polysaccharide derivatives, and phosphazene derivatives (Behrens *et al*. 2014; Beija *et al*. 2011; Chen *et al*. 2013; Elluru *et al*. 2013; Uslu and Güvenaltın 2010).

Up to now, there is few research works published on the subject of temperature-responsive PNPs in the treatment of RA. Researchers are focusing on temperature-response of PNPs aroused by thermo-effect of light-absorption or magnetic action instead of temperature difference between pathological and healthy tissues. However, temperature-responsive PNPs are greatly investigated in anti-cancer therapy, since tumor cells seem to be more sensitive to heat-induced damage than normal cells, which provides sufficient examples and successful new thoughts in the further RA therapy. For instance, Zhang *et al*. successfully prepared a temperature-responsive PEGylated polyaspartamide derivative (mPEG-PAAHP), which exhibited temperature responsiveness obviously around 25 °C. The formulated PTX-loaded nanoparticles showed high drug loading capacity, good stability, and substantial anti-cancer effect against HeLa cells (Zhang and Jiang 2019).

### Photo-responsive PNPs

The strategy of photo-responsive drug delivery is based on light-absorption properties of polymers that undergo phase transition upon light irradiation. Most chemistries respond mainly to ultraviolet (UV), visible, and near-infrared (NIR) lights. These polymeric materials are very attractive for their possibility to control the spatial and temporal triggering of drug release, as well as for their water solubility, biodegradability, and biocompatibility. According to the required application, two main strategies can be used: either a one-time or repeatable on–off drug release. This could be possible due to the ability of some materials to undergo irreversible structural modifications in response to exposure to light, while others can return to the initial state after the trigger is removed. For example, azobenzene group and its derivatives can be reversibly isomerized from *trans*- to *cis*- on ultraviolet-visible light, and from *cis*- to *trans*- by shining light in the visible region, provides release photoregulated control of payload (Lu *et al*. 2008). Some photo-sensitive polymers that are now largely employed in the field of nanomedicine with a potential in the treatment of RA in the future are presented in Table 3. However, only NIR light penetrates more deeply into tissues, which along with low harmful effects to cells, makes it suitable for RA therapy. It is also indicated that RA is characterized by synovial inflammation of the joints within the penetration depth of NIR, which makes possible the applying of a photo-responsive method (Lee *et al*. 2013).

**Table 3:** Examples of photo-sensitive polymeric materials, which can be applied for RA therapy

Polymeric material	Size of nanoparticles (nm)	Stimulus	Applied light wavelength (nm)	Reference
Quinone-methide backbone-diamine spacer 4,5-dime-thoxy-2-nitrobenzyl alcohol	170	Light (self-immolative system)	350; 750	Fomina *et al*. 2010
*o*-nitrobenzyl/methacrylate-functionalized poly(ethylenimine)	160	Light (photolytic degradation)	365	Kim *et al*. 2010
Chitosan hydrochloride 4-oxo-4-(pyren-4-ylmethoxy) butanoic acid	100	Light (photolytic disassembly)	1200	Cui *et al*. 2011
4’-azobenzene dibenzoyl chloride/triethanolamine	20–100	Light (*trans/cis* isomerization)	316	Shen *et al*. 2011
2-nitrophenylethylene glycol/diamine	217–358	Light (photolytic degradation)	550	Lv *et al*. 2012

**Figure 9:**
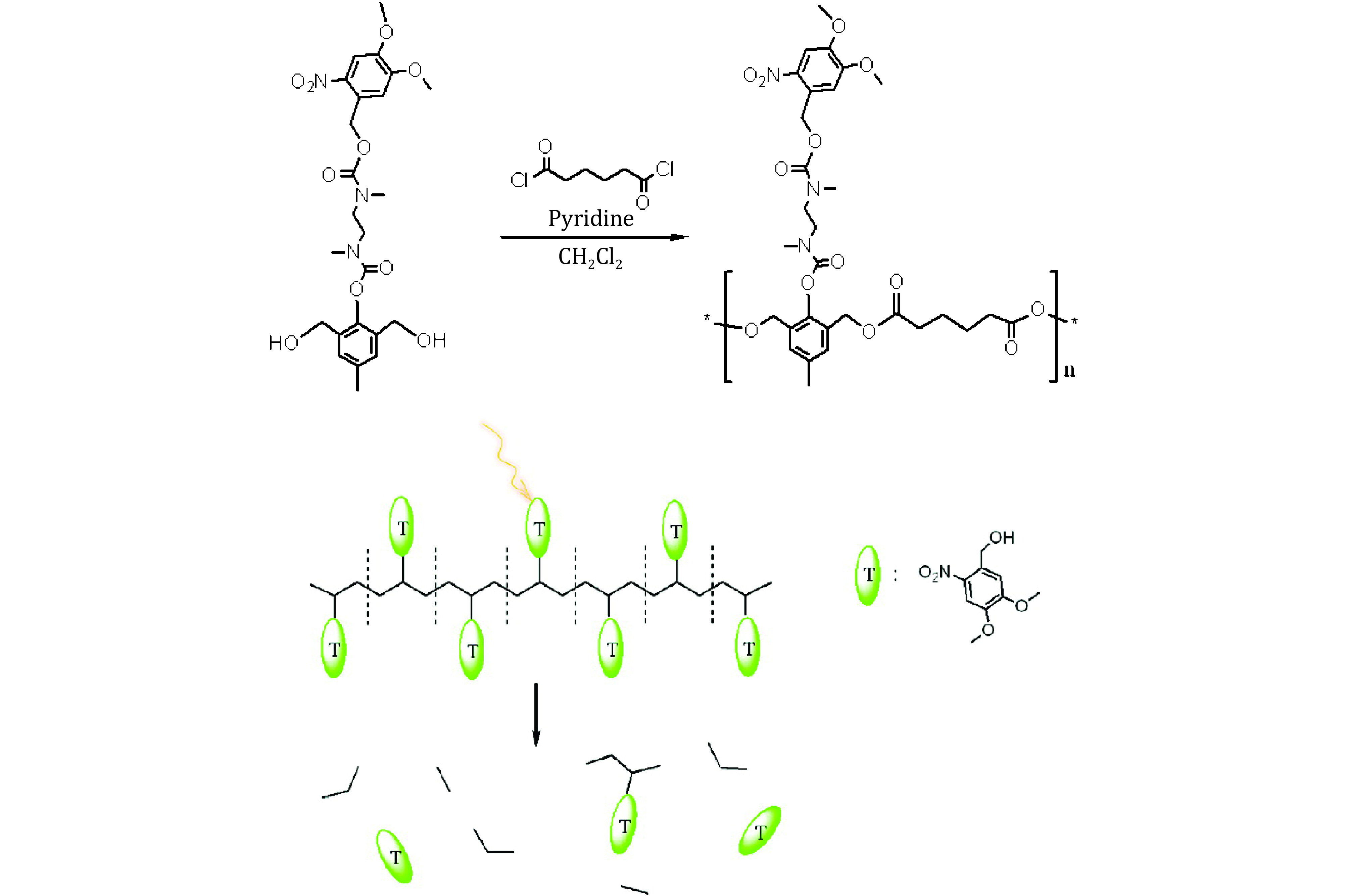
Synthesis route of light-sensitive degradable polymer containing a quinone-methide self-immolative moiety and its subsequent degradation upon irradiation (Fomina *et al*. 2010)

Fomina *et al*. synthesized degradable nanoparticles composed of photo-sensitive polymers with a self-immolative quinone-methide system (Fomina *et al*. 2010). The novel photo-sensitive nanoparticles are capable of controlled triggered burst release of small hydrophobic molecules (Fig. 9). Thus, the versatile design of this system is supposed to allow the triggering group to be sensitive to internal or remote stimuli, which has great potential for RA treatment. In another work, authors reported a polymeric material that was disassembled in response to biological benign levels of NIR irradiation upon two-photon absorption. The design relied on the photolysis of the multiple pendants 4-bromo7-hydroxycoumarin protecting groups, which further triggered a cascade of cyclization and rearrangement reactions leading to the degradation of the polymer backbone (Fomina *et al*. 2011). Furthermore, this polymer is well tolerated by cells both before and after degradation. These results demonstrate that the potential NIR-sensitive polymer may be employed for *in vivo* applications, such as in the treatment of RA. Until now, the versatile design of photo-responsive polymers is under investigation while specific studies on RA in the use of photo-responsive PNPs are greatly insufficient.

### Redox- and enzyme-responsive PNPs

Redox-sensitive nanomaterials are another emerging field for the design of novel drug delivery systems. A great interest in this type of system arises from the difference in redox potential between the oxidizing and reducing space. A number of studies have demonstrated the role of reactive oxygen species (ROS) in the pathogenesis of inflammatory chronic arthropathies, such as RA. ROS are produced mainly during oxidative phosphorylation and can also be generated by activated phagocytic cells during an oxidative burst. They serve as an important intracellular signal that enhances the inflammatory response (Abbas and Monireh 2008; Fonseca *et al*. 2019; Khojah *et al*. 2016). The elevated level of ROS in cancer cells has been widely exploited as the main trigger for the drug release from sensitive delivery systems. For example, the polymers with introduced ROS-sensitive thioketal units or Te-, Se-, B-based linkers in their monomers were used as building blocks for the preparation of responsive nanoparticles. These systems exhibited enhanced selective accumulation at the tumor site and sustained drug release after thioketal groups had been cleaved by ROS (Chen *et al*. 2019a; Jin *et al*. 2019; Wilson *et al*. 2010). The strategies of cancer treatment by ROS-sensitive nanoparticles also can be effectively applied in RA therapy, thus, creating new opportunities to improve the therapeutic outcome by precise control of the system degradation and drug release rate. However, the type of cleavable moiety, its sensitivity, and placement in the delivery system greatly influence the kinetics of payload release. It is also very important to ensure that the degraded units are still biocompatible and can be safely eliminated from the body as non‐toxic fragments.

Enzymes participate in all biological and metabolic processes. They are highly selective and efficient catalysts for organic biochemical reactions. Enzyme-responsive PNPs may provide personalized therapy according to alterations in disease expression in the future. An enhanced expression profile of specific enzymes, such as proteases, phospholipases, or glycosidases observed in pathological situations, such as cancer or inflammation, can be used for enzyme-triggered drug release with an accumulation of drugs at the targeted biological area (De La Rica *et al*. 2012). The design principle of enzyme-mediated nanoparticles is similar to other stimuli, the use of cleavable linkers, to embed them in the polymer backbone and then formulate a drug-loaded carrier. Up to now, some advances in the use of redox- and enzyme-responsive PNPs that are applied in anti-cancer therapy have been reported (Huo *et al*. 2014; Kuang *et al*. 2016). Several redox-responsive polymers have been designed and successfully applied in controlled drug release. However, to the knowledge of the authors, there is no such application in the treatment of RA. Redox- and enzyme-responsive nanomaterials are waiting for being explored in the field of RA. Some potential redox- and enzyme-sensitive polymeric nanomaterials for RA are listed here, which now are being used for anti-cancer therapy (Table 4).

**Table 4:** Examples of potential redox- and enzyme-sensitive polymeric materials, which can be applied for RA therapy

Polymeric material	Size of nanoparticle (nm)	Stimuli	Reference
Poly(ethylene glycol)-b-poly(lactic acid)	374	Redox (disulfide bond)	Song *et al*. 2011
Poly(acrylamide)-poly(methacrylamide)-*N*,*N*’-bis-(acryloyl)cystamine	11.3	Redox (disulfide bond)	Zhao *et al*. 2011
Dimethylaminoethyl methacrylate/*N*,*N*-bis-(acryloyl) cystamine	35	Redox (disulfide bond)	Boyer *et al*. 2009
*N*-(3-aminopropyl) methacrylamide)/acrylamide	20–45	Enzyme (protease-degradable)	Wen *et al*. 2011

### Multistimuli responsive drug delivery

Multistimuli-responsive drug delivery systems are sensitive to more than one stimulus. In the realm of cancer drug delivery, numerous stimuli-responsive carrier systems, in the form of conjugates, dendrimers, liposomes, and micelles, have been developed (Heidarli *et al*. 2017). Although the same potential also exists for RA treatment, few single stimuli drug delivery systems have been reported. In certain pathological conditions, including inflammation or cancer, the coexistence of a pH gradient and an oxidative environment can be observed. Under this conditions pH and redox responsiveness can be used in combination. For instance, conjugation of doxorubicin and antisense-bcl2 oligonucleotide to a four-arm poly(ethylene glycol) with redox-reducible and acid-cleavable linkers resulted in enhanced cancer cell apoptosis (Yoon *et al*. 2013). In another study, a highly packed interlayer-crosslinked micelle with reduction and pH dual sensitivity demonstrated triggered nanocarrier disassembly and a burst of payload release in a reductant-rich environment, resulting in an improvement of the therapeutic index of the loaded drug (Dai *et al*. 2011). Dual responsiveness to pH and temperature of ionically self-assembled nanoparticles and liposomes showed an improved drug release activation (Cui *et al*. 2012; Ta *et al*. 2010). Light-sensitivity can also be combined with pH responsiveness by exploiting the resonance surface properties of palladium and silver (Fang *et al*. 2012). Examples of other systems have demonstrated sensitivity to temperature and magnetic field for MTX delivery (Baeza *et al*. 2012); to light and reducing environment to control the release behavior from block copolymer micelles (Han *et al*. 2012), and to ultrasounds and enzymes to increase drug release from bubble liposomes (Nahire *et al*. 2012).

In addition to pH-, temperature-, photo-, and redox-responsive of nanomaterials, other stimuli from inorganic agents are always combined for the sake of maximizing therapeutic efficiency.

Conventional therapy has a severe drawback of short biological half-life in the development of a drug delivery system. A high dose is needed to assure therapeutic efficiency while it may simultaneously lead to severe side effects. Magnetic nanocarriers can be engineered for local drug delivery at inflammatory sites under the guidance of a magnetic field, providing sufficient therapeutic concentrations with a reduced incidence of unwanted side effects (Polyak and Friedman 2009). Arias *et al*. developed magnetic responsive iron/ethylcellulose (core/shell) nanoparticles loaded with diclofenac sodium for arthritis treatment (Arias *et al*. 2009).

Categories of magnetic core–shell drug carriers are based on a core mainly containing Fe_2_O_3_ and Fe_3_O_4_, and different shells such as poly(lactic-co-glycolic acid), poly(vinylpyrrolidone), chitosan, silica, calcium silicate, metal, and lipids (Albinali *et al*. 2019). Thus, a stimuli-responsive polymer can be as shell encapsulating a magnetic core to obtain more specific targeting and higher release rate. Besides, iron oxide (Fe_3_O_4_) nanoparticles exhibited absorption in the NIR region, which implies their potential role in photothermal therapy. Zhang *et al*. indicated that Fe_3_O_4_, easily fabricated within the size range of 50–400 nm, has a great potential in ablating activated or hyperplastic cells in RA therapy (Zhang *et al*. 2018). Iron oxide is an ideal candidate for investigating the effect of nanoparticles size on distribution and multistimuli treatment of RA.

**Figure 10:**
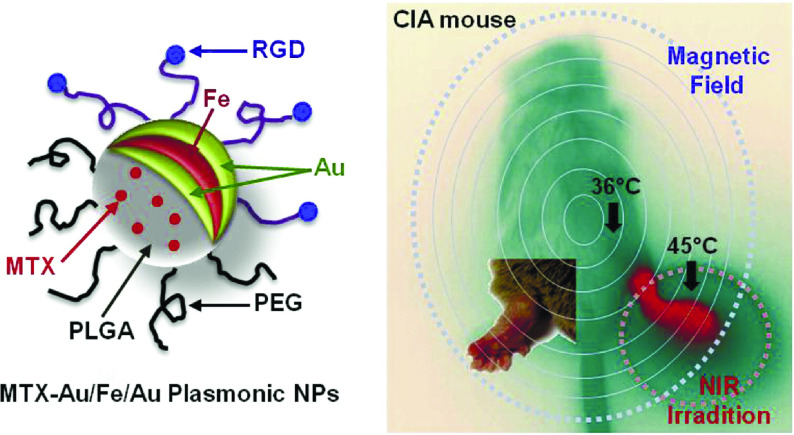
Multifunctional nanoparticle stimulated under NIR irradiation and external magnetic field application. **A** Schematic depiction of a MTX-loaded poly(lactic-co-glycolic acid) (PLGA) gold (Au) / iron (Fe) / gold (Au) half-shell nanoparticles conjugated with target moieties (PEG). **B** Under NIR irradiation, the release of therapeutic agent (MTX) from PLGA accelerated due to local heat which is generated by Au half-shells. And Fe half-shell enables *in vivo* T2-magnetic resonance (MR) imaging in addition to NIR absorbance imaging. Besides, the retention of nanoparticles which have delivered to inflammation region in CIA mice can be enhanced under external magnetic environment (Kim *et al*. 2015)

Gold (Au) is currently capturing huge interests in the field of nanomedicine since gold nanoparticles (Au nanoparticles) demonstrate attractive optical properties, modifiable surface for target molecules or specific biomarkers, and tunable absorption in both visible and NIR regions (Vats *et al*. 2017). Au nanoparticles have various applications including biomolecular ultrasensitive detection, killing cancer cells by hyperthermal treatment, labeling for cells and proteins, and delivering therapeutic agents within cells (Singh *et al*. 2018). Au nanoparticles that absorb NIR and convert it into cytotoxic heat can provide effective photothermal therapeutic effect by accelerating drug release. More importantly, Au nanoparticles are tending to be a multifunctional platform when other stimuli-responsive modes are set. Lima *et al*. designed a novel multi-drug theranostic system for rheumatoid arthritis treatment, where MTX and Au nanoparticles were incorporated into the pegylated-poly(_DL_-lactic-co-glycolic acid) (MTX-PEG-PLGA-Au) nanospheres (Lima and Reis 2015). In their work, *in vitro,* drug release assays revealed that the PEG-PLGA-Au nanospheres could release MTX in a temperature- and pH-responsive mode. In addition, the presence of Au nanoparticles significantly improved the MTX release at photothermal temperature (42°C) and under acidic pH conditions. Kim *et al*. have developed MTX-loaded poly(lactic-co-glycolic acid) (PLGA) Au/Fe/Au half-shell nanoparticles conjugated with arginine–glycine-aspartic acid (Fig. 10), which can be applied for magnetic targeted chemo-photothermal treatment, and *in vivo* multimodal imaging of RA (Kim *et al*. 2015). When combined with consecutive NIR irradiation and external magnetic field application, these nanoparticles provide enhanced therapeutic effects with an MTX dosage of only 0.05% compared with free MTX therapy in the treatment of RA.

## SUMMARY AND PERSPECTIVE

In the past few decades, NMs formulated from stimuli-responsive polymers have made a great contribution to RA treatment. It was proven that the precise drug delivery to the inflammatory joints could significantly improve the therapeutic index and reduce adverse effects associated with the systemic toxicity of anti-RA drugs. The fabrication of NMs sensitive to exogenous or endogenous stimuli may represent an attractive alternative to targeted drug delivery. The wide range of triggers are able to promote the payload release at the desired area and right time, and the variety of responsive materials, which can be assembled in diverse architectures, provide great flexibility in the design of stimuli-responsive systems.

However, despite the significant advancement of stimuli-responsive polymers in the area of drug delivery, there are still a number of concerns that remain to be addressed. Safety issues are considered to be one of the most limiting factors in the medical application. The toxicity of stimuli-responsive systems is multi-factorial and associated with such factors as materials composition, physicochemical features, route of administration, and dosage. Thus, the key point is finding the balance between benefit and risk according to the intended medical use. Furthermore, the lack of degradability or insufficient biocompatibility of many available stimuli-responsive systems significantly reduces their chances of reaching clinics, since it is extremely important that nanocarriers can undergo safe elimination that prevents their accumulation in the body. Another concern is related to the control of the physicochemical properties of nanocarriers’ building blocks that can affect their stimuli-responsiveness. The high sensitivity of these systems to the changes of pH, temperature, or redox potential is not straightforward to achieve, and issues associated with the penetration depth of external stimuli need to be solved. In order to take advantage of nanotechnology and attain greater therapeutic effect, such properties of drug delivery systems as particle size, surface charge, loading capacity, stability during blood circulation, response time, release kinetics, *etc*. should also be taken into account.

Achieving effective drug delivery, avoiding intravenous or intra-articular injections during RA therapy, is now one of the most considerable aspects. Since oral administration is the most convenient and commonly used drug delivery route, it has a crucial importance to find methods to improve the bioavailability of drug delivery systems, and thus, overcome issues associated with invasive administration routes. Moreover, the higher bioavailability along with improved treatment efficacy by drug-bearing nanocarriers can lower the medication amount and administration frequency, which is always being pursued. Transdermal delivery represents an attractive alternative to oral drug delivery in the affected joints. This administration way can be used when there is a significant first-pass drug metabolism in the liver. Moreover, the transdermal delivery can provide drug release for a long period. However, the main drawback is that only a limited number of drugs are suitable for this administration route. For most stimuli-responsive systems, the complex architectural design and difficulties in the scaling-up of their synthesis can hinder their translation to the industrial scale. In this regard, the more facile fabrication of polymer NMs is of importance for further industrial translation. More simple fabrication process and fewer components needed could realize more possibilities of polymer NMs translated to industry.

## Conflict of interest

Yingsi Xie, Ruslan G. Tuguntaev, Cong Mao, Haoting Chen, Ying Tao, Shixiang Wang, Bin Yang and Weisheng Guo declare that they have no conflict of interest.
